# The weak evidence of lip print analysis for sexual dimorphism in forensic dentistry: a systematic literature review and meta-analysis

**DOI:** 10.1038/s41598-021-03680-3

**Published:** 2021-12-17

**Authors:** Ademir Franco, Lorenna Keren Gomes Lima, Murilo Navarro de Oliveira, Walbert de Andrade Vieira, Cauane Blumenberg, Márcio Magno Costa, Luiz Renato Paranhos

**Affiliations:** 1 Centre of Forensic and Legal Medicine and Dentistry, School of Dentistry, University of Dundee, Dundee, Scotland; 2 Division of Forensic Dentistry, Faculdade São Leopoldo Mandic, Campinas, Brazil; 3 Department of Therapeutic Stomatology, Institute of Dentistry, Sechenov University, Moscow, Russia; 4 School of Dentistry, Federal University of Uberlândia, Uberlândia, Brazil; 5 School of Dentistry, Post-Graduate Program in Dentistry, Federal University of Uberlândia, Uberlândia, Brazil; 6 Department of Restorative Dentistry, Endodontics Division, Piracicaba Dental School, State University of Campinas (UNICAMP), Piracicaba, Brazil; 7 Post-Graduate Program in Epidemiology, School of Social Medicine, Federal University of Pelotas, Pelotas, Brazil; 8 Division of Removable Prosthesis and Dental Materials, School of Dentistry, Federal University of Uberlândia, Uberlândia, Brazil; 9 Division of Preventive and Community Dentistry, School of Dentistry, Federal University of Uberlândia, Campus Umuarama, Av. Pará, 1720, Bloco 2G, sala 1, Uberlândia, MG 38405-320 Brazil

**Keywords:** Anatomy, Diagnostic markers

## Abstract

This study aimed to assess the prevalence of lip print patterns among males and females, and to test the diagnostic accuracy of lip pattern analysis for sexual dimorphism in forensic dentistry. A systematic literature review was performed following the PRISMA guidelines. The search was performed in six primary databases and three databases to cover part of the grey literature. Observational and diagnostic accuracy studies that investigated lip print patterns through cheiloscopy for sexual dimorphism were selected. Risk of bias was assessed with the Joanna Briggs Institute (JBI) tool. Proportion meta-analysis using random effects was fitted to pool the accuracy of cheiloscopy. The odds of correctly identifying males and females was assessed through a random effects meta-analysis. GRADE approach was used to assess certainty of evidence. The search found 3,977 records, published between 1982 and 2019. Seventy-two studies fulfilled the eligibility criteria and were included in the qualitative analysis (n = 22,965 participants), and twenty-two studies were sampled for meta-analysis. Fifty studies had low risk of bias. Suzuki and Tsuchihashi’s technique was the most prevalent among studies. The accuracy of sexual dimorphism through cheiloscopy ranged between 52.7 and 93.5%, while the pooled accuracy was 76.8% (95% CI = 65.8; 87.7). There was no difference between the accuracy to identify males or females (OR = 0.71; 95% CI = 0.26; 1.99). The large spectrum of studies on sexual dimorphism via cheiloscopy depicted accuracy percentage rates that rise uncertainty and concern. The unclear performance of the technique could lead to wrong forensic practice.

## Introduction

Cheiloscopy is a field of forensic odontology dedicated to the technical analysis of the human lips^1^. Dating from the 30’s, this procedure is carried out in the context of human identification^2^. More specifically, furrows on the vermillion of the lips are assessed based on their alleged distinctive pattern^3^. In practice, there is speculation about the uniqueness of lip print patterns^4^, ethnical variability^5^ and sexual dimorphism^6^.

Human identification methods must rely on scientifically acceptable tools^7^, such as fingerprint, dental and genetic analyses^8^. Authors of cheiloscopy studies suggest that the analysis of lip prints can support the identification process by narrowing down potential victims based on sex^9^. The contemporary scientific literature on cheiloscopy is vast and growing over time^10–15^. One of the “so-called” advantages of lip prints relies on the alleged unique patterns of furrows that will not repeat between different persons^9^. Authors also claim that lip prints can be found in crime scenes, especially on cigarettes, napkins and glasses^9^. Additionally, the literature points out that most criminals are currently aware of fingerprint analysis and how to avoid leaving such traces in a crime scene—their attention and concern, however, is not the same when it comes to lip prints^9^. Clear-cut furrows that run partially or completely across the lips seem to compose the most prevalent patterns of lip prints, but most of the prevalence studies are restricted to samples that are not even locally representative^4^. Reliable estimates of the presence of lip prints in crime scenes do not exist, but authors progressively endorse this biological trace as “frequent”^8^. Soon, studies on cheiloscopy will populate the scientific literature in forensic science and eventually this technique will be presented in Court as means to collect and analyse evidence. It is the role of science to carry out the scrutiny to (I) test the technique, (II) expose to per review, (III) calculate error rates, (IV) promote standardization, and (V) present to the scientific community to verify whether the technique is acceptable—all steps inherent to Daubert’s standards.

Considering the existing gap reflected by the uncertainty that surrounds the usefulness of lip print patterns and the urgent need to promote evidence-based science, this study was designed to screen the scientific literature with a systematic approach to find out the real value of cheiloscopy for sexual dimorphism. Prevalence rates of lip print patterns and diagnostic accuracy were the targeted as qualitative and quantitative outcomes of interest.

## Materials and methods

### Protocol and registration

This systematic review was performed according to the (1) PRISMA guidelines (Preferred Reporting Items for Systematic Reviews and Meta-Analyses)^16^, (2) the PRISMA standards for Diagnostic Test Accuracy^17^ and (3) the JBI Manual for Evidence Synthesis^18^. The research protocol was submitted for registration at the PROSPERO database.

### Focused question

The systematic review followed the acronym PIRD which stands for population (P), index test (I), reference test (R) and diagnosis of interest (D). The guiding research question was: “Is there evidence to determine the biological sex (diagnosis of interest/reference test) of patients free of pathological and/or genetics changes of the lips (population) using cheiloscopy (index test)?”.

### Eligibility criteria

Only observational (cohort, case–control and cross-sectional) and diagnostic test accuracy studies were included. No restriction was applied regarding the year or language of publication. The exclusion criteria consisted of studies lacking evident information about the technique used for cheiloscopy, cadaver studies and studies with individuals that had genetic/pathologic alterations of the lip.

### Data source and search

The systematic search was performed in August 2020. The primary data sources were Embase, LILACS, PubMed (including MEDLINE), SciELO, Scopus and Web of Science. To avoid/reduce publication bias OpenThesis, OpenGrey and Open Access Theses and Dissertations (OATD) were used as data sources to partially retrieve the grey literature.

Medical Subject Headings (MeSH), Descriptors in Health Sciences (DeCS) and Emtree (Embase Subject Headings) terms were combined by the Boolean operators AND/OR to build search strings (Table 1). Search terms were adapted for each database.

**Table 1:** Strategies for database search.

Database	Search Strategy (August, 2020)
**PubMed** http://www.ncbi.nlm.nih.gov/pubmed	((“Cheiloscopy” OR “Lip Print” OR “Lip Pattern” OR “Lipstick”) AND (“Sex” OR “Gender” OR “Dimorphism”))
**Scopus** http://www.scopus.com/	((“Cheiloscopy” OR “Lip Print” OR “Lip Pattern” OR “Lipstick”) AND (“Sex” OR “Gender” OR “Dimorphism”))
**LILACS** http://lilacs.bvsalud.org/	((“cheiloscopy” OR “lip print” OR “lip pattern” OR “lipstick”) AND (“sex” OR “gender” OR “dimorphism”)) AND (instance:"regional") AND ( db:("LILACS"))
**SciELO** http://www.scielo.org/	((Cheiloscopy OR Lip Print OR Lip Pattern OR Lipstick) AND (Sex OR Gender OR Dimorphism))
**Embase** http://www.embase.com	('Cheiloscopy'/exp OR 'cheiloscopy' OR 'lip print'/exp OR 'lip print' OR 'lip pattern' OR 'lipstick'/exp OR 'lipstick') AND ('sex'/exp OR 'sex' OR 'gender'/exp OR 'gender' OR 'dimorphism'/exp OR 'dimorphism')
**Web Of Science** http://apps.webofknowledge.com/	((“Cheiloscopy” OR “Lip Print” OR “Lip Pattern” OR “Lipstick”) AND (“Sex” OR “Gender” OR “Dimorphism”))
**OpenGrey** http://www.opengrey.eu/	(“Cheiloscopy” OR “Lip Print” OR “Lip Pattern” OR “Lipstick”)
**OpenThesis** http://www.openthesis.org/	“Cheiloscopy”
**Open Access** **Theses and Dissertations (OATD)** https://oatd.org/	“Cheiloscopy”

### Study selection

Initially, studies were identified after a literature search in each of the databases and imported into EndNote Web (Thomson Reuters, Toronto, Canada) (https://www.myendnoteweb.com) software to remove duplicates. Remaining studies were written down in Microsoft Word 2016 (Microsoft Ltd, Washington, USA) to manually remove duplicates. Next, a training exercise was proposed to reviewers to achieve proper agreement during the following phases. The reviewers analyzed 20% of the studies based on the eligibility criteria. The aimed agreement rate was at least 81% (Kappa ≥ 0.81). After training, they were able to perform study selection based on title reading (reviewers were not blind for the authorship and year of publication). The next phase consisted of abstract reading and systematic selection. Studies without abstracts available were not excluded in this phase. Finally, the selected studies underwent full-text reading. Studies excluded in this phase had their reason for exclusion registered separately. During all the study selection process, a third reviewer was enrolled to solve any lack of agreement between the two reviewers.

Studies in which the full text could not be retrieved were requested to the authors by e-mail. Additional support was obtained from the Brazilian Program of Bibliographic Commutation (COMUT) and from the Brazilian Institute of Information on Science and Technology (IBICT). In case of studies published in languages other than English, Portuguese and Spanish, the full text was translated.

### Data extraction

Data extraction was performed by two examiners independently. A template Microsoft Office Excel (Microsoft Ltd, Washington, USA) sheet was used to assure standardized data extraction. The following data were extracted: (I) identifying information—authorship, year and country of publication of the eligible studies; (II) sample profile—size, age interval, sex distribution and geographic region of origin; (III) cheiloscopy-related data—technique used for analysis, general and sex-related lip print patterns, and sensitivity and specificity of cheiloscopy for sexual dimorphism. Data extraction was supervised by a third reviewer and a forensic odontologist.

The corresponding authors were contacted by email (up to three times over two weeks) to obtain relevant information in case of missing or unclear data.

### Risk of bias

The risk of bias and the assessment of individual methodological quality of the eligible studies were accomplished by means of JBI Critical Appraisal tool for observational cross-sectional^19^ or diagnostic test accuracy^20^ studies. Following PRISMA^16^, two reviewers assessed the risk of bias. Lack of agreement between reviewers for any of the questions within the JBI tool was solved by a third examiner.

The percentage of positive answers to the questions led to the final score of the studies. Studies that scored up to 49% of positive answers were classified as “high risk of bias”. Studies with positive answers between 50 and 69% were classified as “moderate risk of bias”, while studies that scored positive answers above 70% were classified as “low risk of bias”.

## Summary measures

The outcomes were explored by means of descriptive analysis and were presented in narrative tables. The prevalence of lip print patterns was reported according to sex and compared between males and females. More specifically, this analysis was performed using a meta-analytical approach of proportions, in which combined prevalence estimates for males and females were estimated using random effects and Freeman-Tukey double transformation to stabilize the model's variances^21^. The heterogeneity between groups was estimated to assess the differences of lip print patterns between males and females. A meta-analysis was adjusted for each combination of lip print pattern, lip side (right/left) and lip position (upper lower). Studies with missing information about lip print pattern, lip side and lip position were not included in the meta-analysis. The meta-analysis was performed separately for the two predominant techniques found in the systematic literature review: Suzuki & Tsuchihasi (1970) and Renaud (1973).

The diagnostic accuracy of the cheiloscopy technique for sexual dimorphism was tested separately for males and females. The absolute number of correct match and mismatch between reference and target lips was extracted from each eligible study and a meta-analysis using random effect was adjusted. To avoid the exclusion of studies that reported zero match or mismatch, a correction of continuity of 0.5 was established in these cases. Studies that provided the number of hits and errors for males and females separately were included in a meta-analysis evaluating if the accuracy of cheiloscopy differed in distinguishing males and females. To assess that, the odds ratio for identifying males compared to females was calculated, and it evaluated if the methods was more or less accurate for sexual dimorphism among males compared to females.

For meta-analyses that included at least 10 studies, publication bias was investigated through Egger’s test by a linear regression of the effect measure on the size of the study^22^. Statistical analyses were performed with Stata version 16.1 (StataCorp LLC, College Station, TX, USA) software. Significance level was set at 5%.

### Certainty of evidence (GRADE approach)

Certainty of evidence and strength of recommendation were assessed with the Grading of Recommendation, Assessment, Development, and Evaluation (GRADE) approach. According to this system, diagnostic accuracy studies start at a high level of certainty and can be downgraded based on risk of bias, inconsistency, indirect evidence, imprecision, and publication bias. The level of certainty among the identified evidence was characterized as high, moderate, low, or very low^23^.

## Results

### Study selection

**Figure 1:**
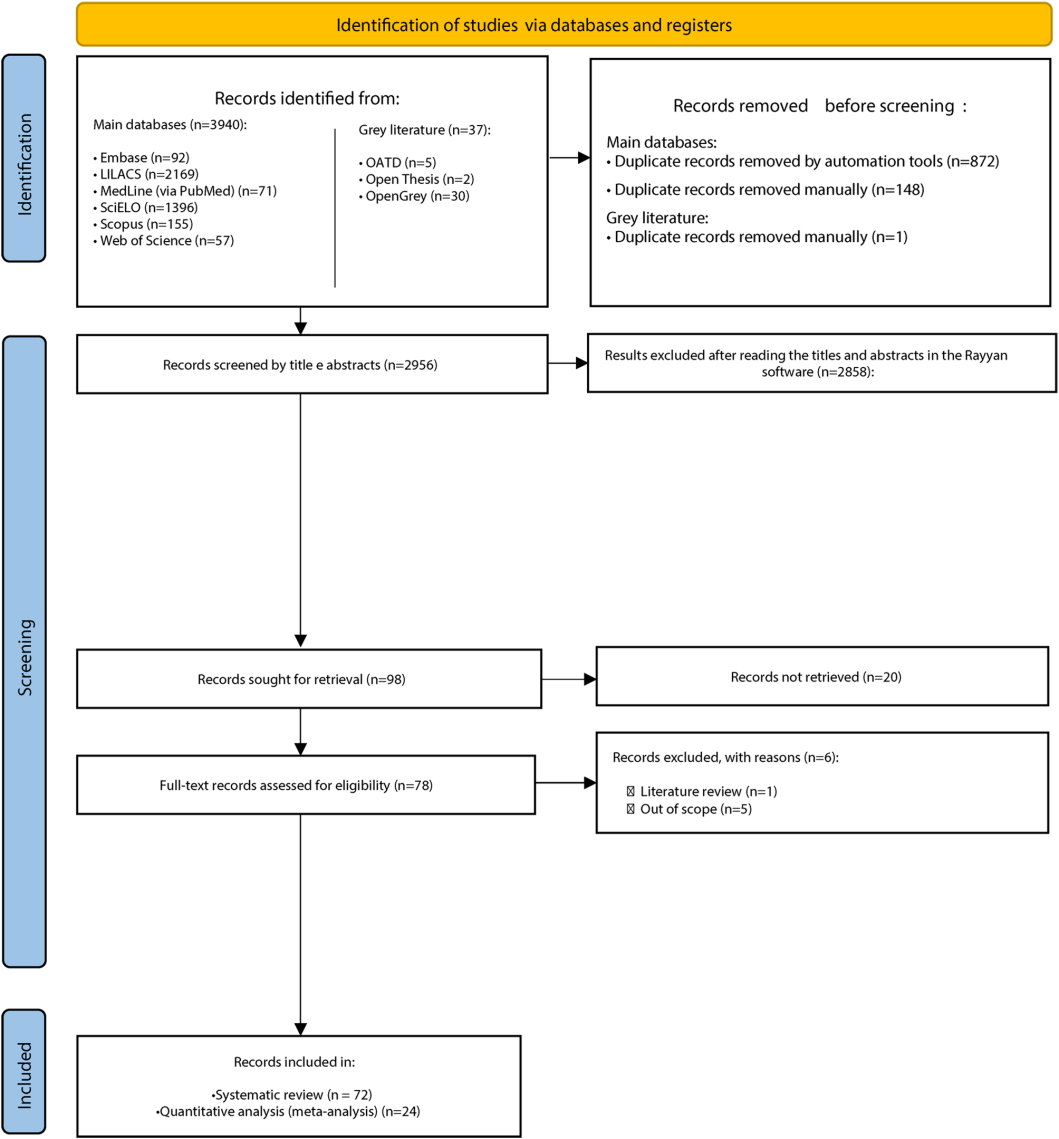
Flowchart diagram, following PRISMA, describing the quantity of studies filtered from identification to the final inclusion in the qualitative and quantitative (meta-) analyses.

The first phase of study selection resulted in 3,977 studies throughout the nine electronic databases. After removing duplicates, the remaining number of studies was 2,956. Exclusions based on title and abstract reading reduced the sample to 98 studies eligible for full-text reading. Six studies did not fulfill the inclusion criteria (Appendix 1), and full texts were not found for twenty studies, even after trying to contact the authors or libraries. Finally, a total of 72 studies were selected for qualitative analysis^1,2,4–6,10–15,24–84^. Quantitative analysis of the accuracy of cheiloscopy for sexual dimorphism included seven studies^1,5,25,48,49,54,80^, and 17 studies^10,11,14,28,30,32,34,36,38,42,43,51,56,60,61,63,82^ were considered in the analyses of the prevalence of lip print patterns (Fig. 1).

### Characteristics of eligible studies

The studies were published between 1982 and 2019, and were from India (n = 52)^1,4–6,10–15,25,27,30,31,35–40,43–46,48–50,53–57,59–62,64,66–75,78,79,81,83,84^, Egypt (n = 3)^2,42,58^, Brazil (n = 3)^26,34,76^, Portugal (n = 2)^32,51^, Pakistan (n = 2)^47,77^, Colombia (n = 2)^29,52^, Nepal (n = 2)^33,82^, France (n = 1)^24^, Iran (n = 1)^63^, Romania (n = 1)^41^, Croatia (n = 1)^65^, Saudi Arabia (n = 1)^28^ and Poland (n = 1)^80^. The total sample of participants across studies was 22,965. The age interval of the of participants ranged from 1 to 83 years (Table 2). Fourteen studies did not describe the ethical aspects adopted in the study. None of the cross-sectional studies reported STROBE checklist as the guideline of choice.

**Table 2:** Main characteristics of eligible studies.

Authors, year^ref^	Country	Age (years)	n	Technique	Data collection
Fauvel et al., 1982^24^	France	3–73	111 (42♂;69♀)	Fauvel’s	Polymer and varnish
Sonal et al., 2005^25^	India	19–29	50 (20♂;30♀)	Suzuki and Tsuchihashi’s	Lipstick and paper
Barros, 2006^26^	Brazil	n/r	120 (60♂;60♀)	Suzuki and Tsuchihashi’s	Lipstick, paper and photographs
Augustine et al., 2008^10^	India	3–83	600 (280♂;320♀)	Suzuki and Tsuchihashi’s	Lipstick and paper digitized
Sharma and Saxena, 2009^27^	India	n/r	100 (50♂;50♀)	Suzuki and Tsuchihashi’s	Lipstick and paper
El Domiaty et al., 2010^28^	Saudi Arabia	18–40	966 (426♂;540♀)	Renaud’s	Lipstick, paper and photographs
Chalapud et al., 2011^29^	Colombia	17–30	47 (23♂;24♀)	Renaud’s	Lipstick, paper and photographs
Gupta et al., 2011^30^	India	18–30	146 (73♂;73♀)	Suzuki and Tsuchihashi’s	Lipstick and paper
Prasad and Vanishree, 2011^31^	India	17–21	100 (50♂;50♀)	Suzuki and Tsuchihashi’s	Lipstick and paper
Amith et al., 2012^11^	India	10–25	1539 (695♂;844♀)	Suzuki and Tsuchihashi’s	Lipstick and paper
Babladi et al., 2012^12^	India	18–22	124 (66♂;58♀)	Suzuki and Tsuchihashi’s	Lipstick and paper
Costa and Caldas, 2012^32^	Portugal	20–33	50 (25♂;25♀)	Suzuki and Tsuchihashi’s	Lipstick and paper digitized
Karki, 2012^33^	Nepal	18–25	150 (75♂;75♀)	Suzuki and Tsuchihashi’s	Lipstick and paper
Oliveira et al., 2012^34^	Brazil	n/r	104 (54♂;50♀)	Suzuki and Tsuchihashi’s	Lipstick, paper and photographs
Prabhu et al., 2012^35^	India	19–28	100 (♂♀n/r)	Suzuki and Tsuchihashi’s	Lipstick, paper and scanning
Rastogi and Parida, 2012^36^	India	18–25	100 (♂♀n/r)	Suzuki and Tsuchihashi’s	Lipstick and paper
Vats et al., 2012^37^	India	8–60	1399 (781♂;618♀)	Suzuki and Tsuchihashi’s	Lipstick and paper
Bansal et al., 2013^6^	India	20–50	5000 (2500♂;2500♀)	Suzuki and Tsuchihashi’s	Lipstick and paper
Kautilya et al., 2013^38^	India	18–25	100 (50♂;50♀)	Suzuki and Tsuchihashi’s	Lipstick and paper
Koneru et al., 2013^39^	India	18–21	60 (30♂;30♀)	Suzuki and Tsuchihashi’s	Lipstick and paper
Padmavathi et al., 2013^40^	India	n/r	250 (♂♀)	Suzuki and Tsuchihashi’s	Lipstick, paper and photographs
Popa et al., 2013^41^	Romania	24–37	100 (50♂;50♀)	Suzuki and Tsuchihashi’s	Lipstick and paper
Ragab et al., 2013^42^	Egypt	2–65	955 (235♂;720♀)	Renaud’s	Lipstick, paper and scanning
Sekhon et al., 2013^43^	India	n/r	300 (100♂;200♀)	Suzuki and Tsuchihashi’s	Lipstick, paper and scanning
Verma et al., 2013^44^	India	18–25	208 (85♂;123♀)	Suzuki and Tsuchihashi’s	Lipstick and paper
Gupta et al., 2014^45^	India	18–30	378 (189♂;189♀)	Suzuki and Tsuchihashi’s	Lipstick and paper
Hammad et al., 2014^46^	Pakistan	19–25	100 (30♂;70♀)	Suzuki and Tsuchihashi’s	Lipstick and paper
Multani et al., 2014^47^	India	15–55	200 (100♂;100♀)	Suzuki and Tsuchihashi’s	Lipstick and paper
Nagalaxmi et al., 2014^48^	India	20–30	60 (30♂;30♀)	Suzuki and Tsuchihashi’s	Lipstick and paper
Ramaligam et al., 2014^49^	India	20–30	40 (20♂;20♀)	Suzuki and Tsuchihashi’s	Lipstick and paper
Sharma et al., 2014^5^	India	17–26	200 (100♂;100♀)	Suzuki and Tsuchihashi’s	Lipstick and paper
Abidullah et al., 2015^13^	India	18–30	200 (100♂;100;♀)	Suzuki and Tsuchihashi’s	Lipstick and paper
Bharathi and Thenmozhi, 2015^51^	India	n/r	100 (24♂;76♀)	Suzuki and Tsuchihashi’s	Lipstick and paper
Cartaxo, 2015^52^	Portugal	17–40	202 (94♂;108♀)	Suzuki and Tsuchihashi’s	Lipstick, paper and photographs
Hernández et al., 2015^53^	Colombia	18–25	60 (30♂;30♀)	Suzuki and Tsuchihashi’s	Lipstick and paper
Kaul et al., 2015^54^	India	1–80	755 (375♂;380♀)	Suzuki and Tsuchihashi’s	Lipstick and paper
Nagpal et al., 2015^55^	India	18–24	60 (20♂;40♀)	Suzuki and Tsuchihashi’s	Lipstick and paper
Peeran et al., 2015^56^	India	18–35	104 (37♂;67♀)	Suzuki and Tsuchihashi’s	Lipstick and paper
Shah and Jayaraj, 2015^57^	India	17–25	200 (100♂;100♀)	Suzuki and Tsuchihashi’s	Lipstick and paper
Sharma et al., 2015^58^	India	18–25	201 (107♂;94♀)	Suzuki and Tsuchihashi’s	Lipstick and paper
Badiye and Kapoor, 2016^50^	India	18–25	400 (200♂;200♀)	Suzuki and Tsuchihashi’s	Lipstick and photographs
Aziz et al., 2016^59^	Egypt	n/r	120 (60♂;60♀)	Suzuki and Tsuchihashi’s	Lipstick and paper
Borase et al., 2016^60^	India	20–50	496 (326♂;170♀)	Renaud’s	Lipstick and paper digitized
Jeergal et al., 2016^61^	India	18–60	200 (100♂;100♀)	Suzuki and Tsuchihashi’s	Lipstick and paper digitized
Krishnan et al., 2016^62^	India	18–21	60 (30♂;30♀)	Suzuki and Tsuchihashi’s	Lipstick and paper
Moshfeghi et al., 2016^63^	Iran	13–70	96 (22♂;74♀)	Suzuki and Tsuchihashi’s	Lipstick and paper
Negi and Negi, 2016^64^	India	n/r	200 (100♂;100♀)	Nagasupriya’s	Lipstick and paper
Simovic et al., 2016^65^	Croatia	n/r	90 (40♂;50♀)	Suzuki and Tsuchihashi’s	Lipstick and paper
Tarvadi and Goyal, 2016^66^	India	18–25	100 (50♂;50♀)	Suzuki and Tsuchihashi’s	Lipstick and paper
Alzapur et al., 2017^4^	India	17–19	100 (50♂;50♀)	Suzuki and Tsuchihashi’s	Lipstick and paper
Basheer et al., 2017^14^	India	18–30	858 (471♂;387♀)	Suzuki and Tsuchihashi’s	Lipstick and paper
Kumar, 2017^67^	India	10–16	200 (100♂;100♀)	Suzuki and Tsuchihashi’s	Lipstick and paper
Chaudhari et al., 2017^68^	India	25–50	150 (75♂75♀)	Suzuki and Tsuchihashi’s	Lipstick and paper
Gouda and Rao, 2017^69^	India	18–23	100 (50♂50♀)	Suzuki and Tsuchihashi’s	Lipstick and paper
Kapoor and Badyie, 2017^70^	India	18–25	200 (100♂100♀)	Suzuki and Tsuchihashi’s	Lipstick and photographs
Naik et al., 2017^71^	India	18–20	100 (50♂;50♀)	Suzuki and Tsuchihashi’s	Lipstick and Whatman paper filter
Sandhu et al., 2017^72^	India	18–30	1200 (540♂;660♀)	Suzuki and Tsuchihashi’s	Lipstick and paper
Tandon et al., 2017^73^	India	20–50	100 (50♂;50♀)	Suzuki and Tsuchihashi’s	Lipstick and paper
Vignesh et al., 2017^74^	India	3–6	300 (♂♀ n/r)	Suzuki and Tsuchihashi’s	Lipstick and paper
Ahmed et al., 2018^2^	Egypt	26.8 ± 10.4	221 (105♂;116♀)	Suzuki and Tsuchihashi’s	Lipstick and paper
Bai et al., 2018^15^	India	18–25	300 (150♂;150♀)	Suzuki and Tsuchihashi’s	Lipstick and paper
Herrera et al., 2018^75^	Brazil	18–71	50 (25♂;25♀)	Suzuki and Tsuchihashi’s	Lipstick, CD, glass and photographs
Ishaq et al., 2018^76^	Pakistan	n/r	250 (125♂;125♀)	Suzuki and Tsuchihashi’s	Lipstick and paper
Manikya et al., 2018^77^	India	18–23	180 (90♂;90♀)	Suzuki and Tsuchihashi’s	Lipstick and paper
Bhagyashree et al., 2018^78^	India	18–30	100 (50♂;50♀)	Suzuki and Tsuchihashi’s	Lipstick, paper and glass
Thomas et al., 2018^79^	India	18–26	128 (67♂;61♀)	Suzuki and Tsuchihashi’s	Lipstick and paper
Topczyłko et al., 2018^80^	Poland	15–30	242 (76♂;166♀)	Suzuki and Tsuchihashi’s, Renaud’s, Vahanwala’s	n/r
Bansal et al., 2019^1^	India	18–21	200 (100♂;100♀)	Suzuki and Tsuchihashi’s	Lipstick, paper, glass and powder
Divyadharsini and Kumar, 2019^81^	India	20–30	100 (50♂;50♀)	Suzuki and Tsuchihashi’s	Lipstick and paper
Gurung et al., 2019^82^	Nepal	17–24	205 (141♂;64♀)	Suzuki and Tsuchihashi’s	Lipstick and paper
Vaishnavi et al., 2019^83^	India	15–20	50 (25♂;25♀)	n/r	Lipstick and paper
Yendriwati et al., 2019^84^	India	20–26	30 (15♂;15♀)	Suzuki and Tsuchihashi’s	Lipstick and paper

♂: Male ♀: Female; n/r: Not reported by the authors.

Sixty-four studies^1,2,4–6,10–15,25–27,30–41,43–59,61–63,65–79,81,82,84^ used the technique of Suzuki and Tsuchihashi (1970), four studies^28,29,42,60^ used Renaud’s (1973) technique, one study^24^ used Fauvel’s (1985) technique, one study^64^ used Nagasupriya’s (2011) technique, and one study^80^ combined the techniques of Suzuki and Tsuchihashi (1970), Renaud (1973), and Vahanwala (2000). One study^83^ did not report which technique was used. In general, twenty-four studies (33%)^12,14,24,26,28,31,35,42–44,47,51,53–55,57,63,66,67,74–76,82^ did not find evidence of difference of lip print patterns between males and females, while 67%^1,2,4–6,10,11,13,15,25,27,29,31–34,36–41,45,46,48–50,52,56,58–62,64,65,68–73,77–81,83,84^ detected differences.

### Individual risk of bias

Fifty eligible studies^2,4,5,10,14,25,26,28–30,32,35,38–40,42,44–46,49–58,60–63,66–72,74,76–79,81–84^ had low risk of bias, while 22 studies^1,6,11–13,15,24,27,31,33,36,37,41,43,47,48,59,64,65,73,75,80^ had moderate risk of bias (Tables 3 and 4). All the questions in JBI tool for cross-sectional studies were applicable, while three questions were not applicable in the JBI tool for diagnostic test accuracy studies.

**Table 3:** Risk of bias assessed by the Joanna Briggs Institute Critical Appraisal Tools for use in JBI Critical Appraisal Checklist for Diagnostic Accuracy Studies.

Study	Q.1	Q.2	Q.3	Q.4	Q.5	Q.6	Q.7	Q.8	Q.9	Q.10	% Yes	Risk
Sonal et al., 2005^25^	–	–	✓	✓	N/A	✓	N/A	N/A	✓	✓		Low
Nagalaxmi et al., 2014^48^	–	–	✓	U	N/A	✓	N/A	N/A	✓	✓		Moderate
Ramaligam et al., 2014^49^	–	–	✓	✓	N/A	✓	N/A	N/A	✓	✓		Low
Sharma et al., 2014^5^	U	–	✓	✓	N/A	✓	N/A	N/A	✓	✓		Low
Kaul et al., 2015^54^	U	–	✓	✓	N/A	✓	N/A	N/A	✓	✓		Low
Topczyłko et al., 2018^80^	U	–	✓	U	N/A	✓	N/A	N/A	✓	✓		Moderate
Bansal et al., 2019^1^	U	–	✓	U	N/A	✓	N/A	N/A	✓	✓		Moderate

Q.1. Was a consecutive or random sample of patients enrolled? Q.2. Was a case control design avoided? Q. 3. Did the study avoid inappropriate exclusions? Q.4. Were the index test results interpreted without knowledge of the results of the reference standard? Q.5. If a threshold was used, was it pre-specified? Q.6. Is the reference standard likely to correctly classify the target condition? Q.7. Were the reference standard results interpreted without knowledge of the results of the index test? Q.8. Was there an appropriate interval between index test and reference standard? Q.9. Did all patients receive the same reference standard? Q.10. Were all patients included in the analysis? ✓: Yes; –: No; U : Unclear: N/A: Not applicable.

**Table 4:** Risk of bias assessed by the Joanna Briggs Institute Critical Appraisal Tools for use in JBI Critical Appraisal Checklist for Analytical Cross Sectional Studies.

Authors	Q1	Q2	Q3	Q4	Q5	Q6	Q7	Q8	% Yes	Risk
Fauvel et al., 1982^24^	✓	✓	✓	✓	–	–	–	✓	62.5	Moderate
Barros, 2006^26^	✓	✓	✓	✓	✓	✓	–	✓	87.5	Low
Augustine et al., 2008^10^	–	✓	✓	✓	–	–	✓	✓	62.5	Moderate
Sharma and Saxena, 2009^27^	✓	✓	✓	✓	–	–	–	✓	62.5	Moderate
El Domiaty et al., 2010^28^	✓	✓	✓	✓	–	–	✓	✓	75	Low
Chalapud et al., 2011^29^	–	✓	✓	✓	✓	–	✓	✓	75	Low
Gupta et al., 2011^30^	✓	✓	✓	✓	✓	✓	–	✓	87.5	Low
Prasad and Vanishree, 2011^31^	–	✓	✓	✓	–	–	–	✓	50	Moderate
Amith et al., 2012^11^	✓	✓	✓	✓	–	–	–	✓	62.5	Moderate
Babladi et al., 2012^12^	✓	✓	✓	✓	–	–	–	✓	62.5	Moderate
Costa and Caldas, 2012^32^	✓	✓	✓	✓	✓	✓	✓	✓	100	Low
Karki, 2012^33^	–	✓	✓	✓	–	–	–	✓	50	Moderate
Oliveira et al., 2012^34^	✓	–	✓	✓	✓	✓	✓	✓	87.5	Low
Prabhu et al., 2012^35^	✓	✓	✓	✓	✓	✓	–	✓	87.5	Low
Rastogi and Parida, 2012^36^	✓	✓	✓	✓	–	–	–	✓	62.5	Moderate
Vats et al., 2012^37^	–	–	✓	✓	–	–	✓	✓	50	Moderate
Bansal et al., 2013^6^	–	–	✓	✓	–	✓	–	✓	50	Moderate
Kautilya et al., 2013^38^	✓	✓	✓	✓	–	–	✓	✓	75	Low
Koneru et al., 2013^39^	✓	✓	✓	✓	✓	✓	–	✓	87.5	Low
Padmavathi et al., 2013^40^	–	✓	–	✓	–	–	✓	✓	75	Low
Popa et al., 2013^41^	✓	✓	✓	✓	–	–	–	✓	62.5	Moderate
Ragab et al., 2013^42^	✓	✓	✓	✓	✓	✓	✓	✓	100	Low
Sekhon et al., 2013^43^	✓	✓	✓	✓	–	–	–	✓	62.5	Moderate
Verma et al., 2013^44^	✓	✓	✓	✓	–	–	✓	✓	75	Low
Gupta et al., 2014^45^	✓	✓	✓	✓	✓	✓	✓	✓	100	Low
Hammad et al., 2014^46^	✓	✓	✓	✓	–	–	–	✓	62.5	Moderate
Multani et al., 2014^47^	✓	✓	✓	✓	✓	✓	✓	✓	100	Low
Abidullah et al., 2015^13^	✓	✓	✓	✓	✓	✓	–	✓	87.5	Low
Bharathi and Thenmozhi, 2015^51^	✓	–	✓	✓	✓	✓	–	✓	75	Low
Cartaxo, 2015^52^	✓	✓	✓	✓	✓	✓	✓	✓	100	Low
Hernández et al., 2015^53^	✓	✓	✓	✓	–	–	✓	✓	75	Low
Nagpal et al., 2015^55^	✓	✓	✓	✓	✓	✓	✓	✓	100	Low
Peeran et al., 2015^56^	✓	✓	✓	✓	✓	✓	–	✓	87.5	Low
Shah and Jayaraj, 2015^57^	✓	✓	✓	✓	✓	✓	✓	✓	100	Low
Sharma et al., 2015^58^	✓	✓	✓	✓	✓	✓	–	✓	87.5	Low
Badiye and Kapoor, 2016^50^	–	✓	✓	✓	–	–	✓	✓	62.5	Moderate
Aziz et al., 2016^59^	✓	✓	✓	✓	✓	✓	✓	✓	100	Low
Borase et al., 2016^60^	✓	✓	✓	✓	✓	✓	✓	✓	100	Low
Jeergal et al., 2016^61^	✓	–	✓	✓	✓	✓	✓	✓	87.5	Low
Krishnan et al., 2016^62^	✓	✓	✓	✓	✓	✓	✓	✓	100	Low
Moshfeghi et al., 2016^63^	✓	✓	✓	✓	✓	✓	✓	✓	100	Low
Negi and Negi, 2016^64^	–	–	✓	✓	✓	✓	–	✓	62.5	Moderate
Simovic et al., 2016^65^	–	–	✓	✓	–	–	✓	✓	50	Moderate
Tarvadi and Goyal, 2016^66^	✓	✓	✓	✓	✓	✓	–	✓	87.5	Low
Alzapur et al., 2017^4^	✓	✓	✓	✓	✓	✓	✓	✓	100	Low
Basheer et al., 2017^14^	✓	✓	✓	✓	–	–	–	✓	62.5	Moderate
Kumar, 2017^67^	✓	✓	✓	✓	✓	✓	✓	✓	100	Low
Chaudhari et al., 2017^68^	✓	✓	✓	✓	✓	✓	–	✓	87.5	Low
Gouda and Rao, 2017^69^	✓	✓	✓	✓	✓	✓	–	✓	87.5	Low
Kapoor and Badyie, 2017^70^	✓	✓	✓	✓	✓	✓	✓	✓	100	Low
Naik et al., 2017^71^	–	✓	✓	✓	✓	✓	–	✓	75	Low
Sandhu et al., 2017^72^	✓	✓	✓	✓	✓	✓	✓	✓	100	Low
Tandon et al., 2017^73^	–	✓	✓	✓	–	–	–	✓	50	Moderate
Vignesh et al., 2017^74^	✓	✓	✓	✓	✓	✓	✓	✓	100	Low
Ahmed et al., 2018^2^	✓	✓	✓	✓	✓	✓	–	✓	87.5	Low
Bai et al., 2018^15^	✓	✓	✓	✓	✓	✓	✓	✓	100	Low
Herrera et al., 2018^75^	✓	✓	✓	✓	✓	✓	✓	✓	100	Low
Ishaq et al., 2018^76^	–	✓	✓	✓	✓	✓	✓	✓	87.5	Low
Manikya et al., 2018^77^	✓	✓	✓	✓	✓	✓	✓	✓	100	Low
Bhagyashree et al., 2018^78^	–	–	✓	✓	–	–	✓	✓	50	Moderate
Thomas et al., 2018^79^	✓	✓	✓	✓	✓	✓	✓	✓	100	Low
Divyadharsini and Kumar, 2019^81^	✓	✓	✓	✓	✓	✓	✓	✓	100	Low
Gurung et al., 2019^82^	✓	✓	✓	✓	✓	✓	✓	✓	100	Low
Vaishnavi et al., 2019^83^	✓	✓	✓	✓	✓	✓	–	✓	87.5	Low
Yendriwati et al., 2019^84^	✓	✓	✓	✓	–	–	✓	✓	75	Low

Q1. Were the criteria for inclusion in the sample clearly defined? Q2. Were the study subjects and the setting described in detail? Q3. Was the exposure measured in a valid and reliable way? Q4. Were objective, standard criteria used for measurement of the condition? Q5. Were confounding factors identified? Q6. Were strategies to deal with confounding factors stated? Q7. Were the outcomes measured in a valid and reliable way? Q8. Was appropriate statistical analysis used? ✓: Yes; –: No; U : Unclear: N/A: Not applicable.

Regarding cross-sectional studies, questions #5 and #6 had a negative answer in 25 studies^6,11–13,15,24,26,27,30,31,33,35,36,39,41,43,47,50,55,57,64,66–68,71,73,83^. These questions verify if the study identified and avoided confounding factors, since studies should minimize the risk of bias describing factors that could influence on the process of collecting lip print evidence. In 28 studies^2,6,10,11,13,15,24,26,27,30,31,33,35,36,39,41,43,47,50,55,57,64,66–68,71,73,83^ question #7 had a negative answer. This question has a direct impact in the quality of the evidence because it verifies if the outcomes were obtained in a reliable way. An example of attitude towards a positive answer is the minimization of bias by describing the process of intra- and inter-examiner training.

Concerning diagnostic test accuracy studies, questions #1 and #2 were marked as ‘unclear’ or ‘no’ for all studies^1,5,25,48,49,54,80^. The first question checked whether the sample was selected consecutively or randomly. The second question was related to the methodological design of the studies; all studies recruited participants that were already known, by other means, to have the diagnosis of interest and investigated whether the test of interest correctly identified them as such. Moreover, question 4 was marked as 'unclear' for three studies^1,48,80^ that did not provide details regarding blindness of the index test.

### Synthesis of results

#### Primary outcome—accuracy for sexual dimorphism

**Figure 2:**
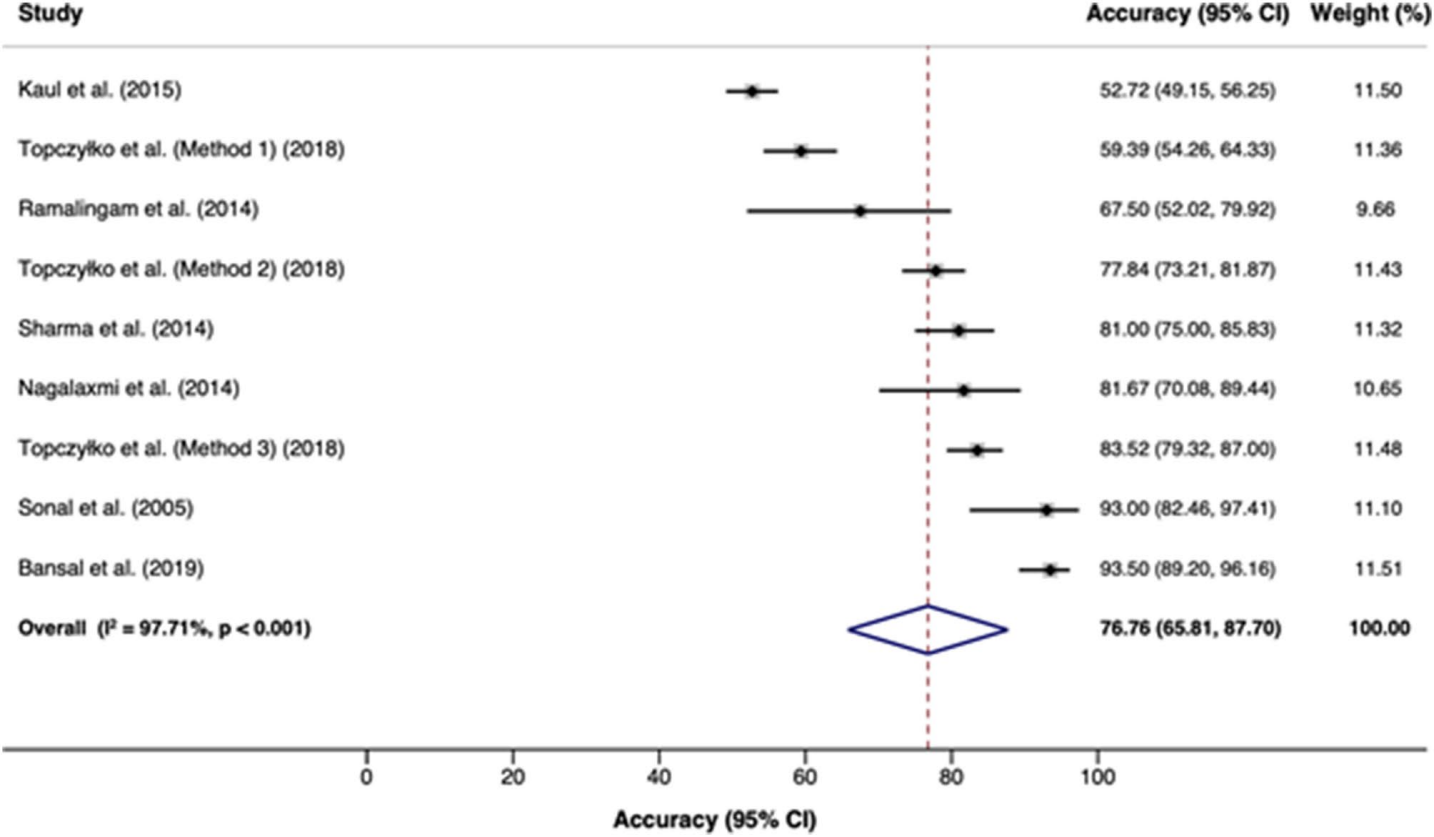
Overall compilation of accuracy rates across seven eligible studies that reported the sufficient data for quantitative analysis.

Seven studies^1,4,25,48,49,54,80^ were included in the meta-analysis of the accuracy of lip prints for sexual dimorphism. Out of the seven studies, nine accuracy assessments were included in the meta-analysis—since the study by Topczyłko et al.^80^ evaluated three different methods. The overall accuracy was 76.8% (95% CI = 65.8; 87.7, I^2^ = 97%) (Fig. 2). Individual accuracy rates ranged from 52.7 to 93.5%.

**Figure 3:**
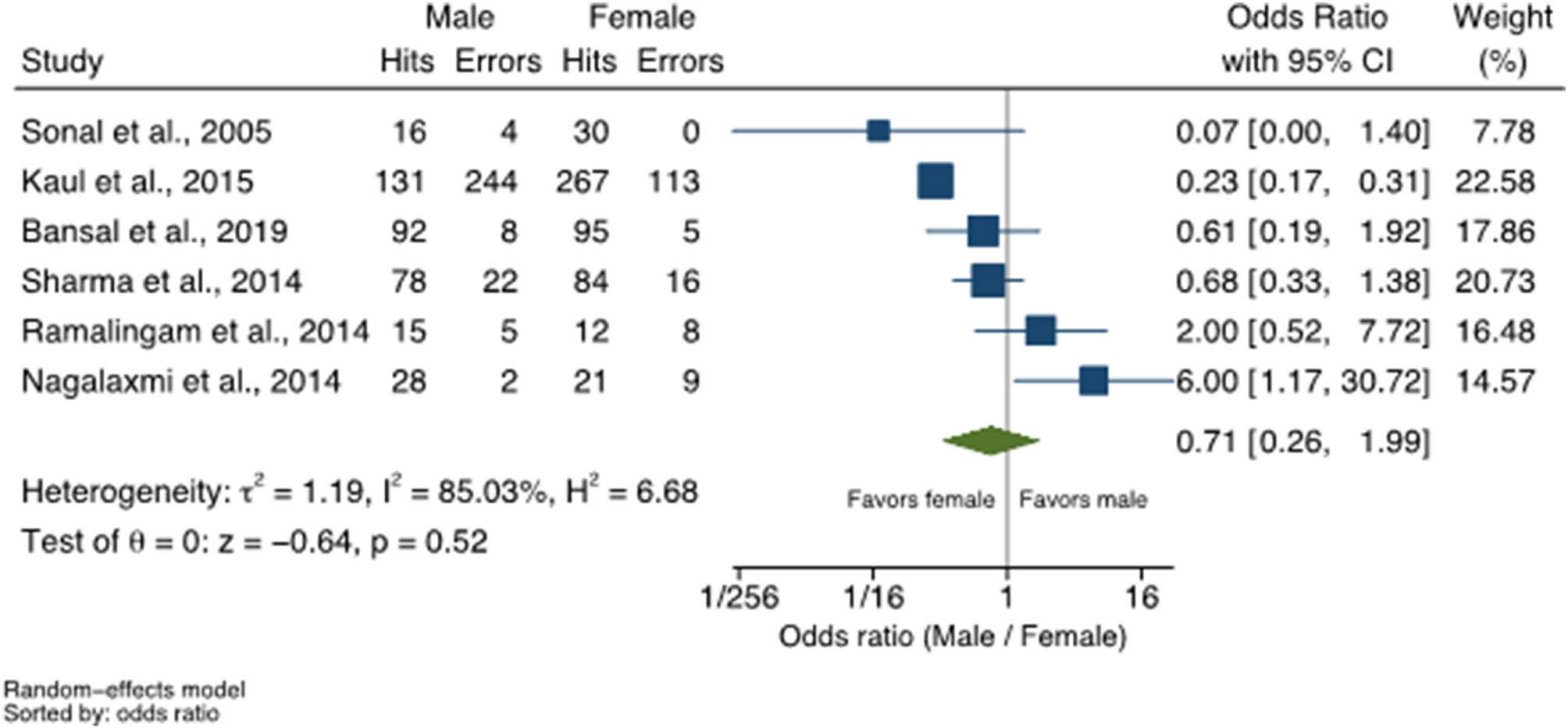
Odds ratio depicting the accuracy of cheiloscopy for distinguishing males from females. Random-effects model applied within six eligible studies.

Six out of the seven studies included in accuracy meta-analysis provided the number of hits and error according to the sex of the patient and were included in a meta-analysis that assessed if the odds of distinguishing males was different compared to the odds of distinguishing females. Overall, there were no differences to diagnose males compared to females (OR = 0.71; 95% CI = 0.26; 1.99, I^2^ = 85%). Only specific studies, such as Kaul et al. (2015)^53^ and Nagalaxmi et al. (2014)^48^, described differences for sexual dimorphism (Fig. 3). The first showed 77% higher odds of identifying females compared to males (OR = 0.23; 95% CI = 0.27; 0.31), while the second showed sixfold higher odds of identifying males compared to females (OR = 6.00; 95% CI = 1.17; 30.72). One study^80^ did not report samples divided by sex and was not included in the analysis.

#### Secondary outcome—prevalence of lip prints

According to the technique of Suzuki and Tsuchihashi (1970), lip print pattern type 2 was the most prevalent (> 30%), while type 5 was the rarest pattern (< 3%) (Table 5). Sex differences based on prevalence rates were not detected. Publication bias was identified for studies analyzing lip print type 1’ for the upper and lower dental arches on the right side, for lip print type 4 for the upper arch on the left and right sides, and for lip print type 4 for the lower arch on the right side.

**Table 5:** Lip pattern prevalence according to sex and dental arch for Suzuki and Tsuchihashi’s method for cheiloscopy classification.

	Left side using Suzuki and Tsuchihashi's (n = 14)^¶^			Right side using Suzuki and Tsuchihashi's (n = 14)^¶^		
	Male	Female	P value	Male	Female	P value
**Upper dental arch**						
Type 1	16.3 (11.8; 21.4)	16.0 (11.2; 21.4)	0.892	18.2 (13.2; 23.7)	17.2 (11.7; 23.3)	0.778
Type 1'	12.4 (6.6; 19.6)	12.8 (6.9; 20.0)	0.964	12.6 (6.7; 20.0)	12.3 (6.4; 19.9)	0.928*
Type 2	23.7 (20.9; 26.6)	25.7 (21.6; 30.0)	0.473	23.7 (20.9; 26.6)	25.7 (21.6; 30.0)	0.473
Type 3	23.8 (17.8; 30.4)	18.0 (11.1; 26.1)	0.246	23.8 (17.8; 30.4)	18.0 (11.1; 26.1)	0.246
Type 4	10.2 (6.7; 14.1)	13.0 (7.8; 19.4)	0.454*	10.2 (6.7; 14.1)	13.0 (7.6; 19.4)	0.454*
Type 5	3.7 (1.3; 6.9)	3.5 (1.1; 6.9)	0.863	3.7 (1.3; 6.9)	3.5 (1.1; 6.9)	0.863
**Lower dental arch**						
Type 1	19.7 (10.4; 30.9)	24.1 (13.7; 36.3)	0.580	23.7 (14.1; 34.9)	25.0 (14.9; 36.8)	0.875
Type 1'	10.2 (5.4; 16.3)	11.3 (6.3; 17.5)	0.816	10.2 (5.4; 16.3)	11.3 (6.3; 17.5)	0.816*
Type 2	31.7 (20.0; 44.7)	31.4 (22.3; 41.2)	0.955	31.7 (20.0; 44.7)	31.4 (22.3; 41.2)	0.955
Type 3	18.2 (8.5; 30.4)	12.5 (4.6; 23.3)	0.435	18.2 (8.5; 30.4)	12.5 (4.6; 23.3)	0.435
Type 4	5.6 (2.6; 9.4)	5.2 (1.9; 9.6)	0.822	5.6 (2.6; 9.4)	5.2 (1.9; 9.6)	0.822*
Type 5	2.5 (0.8; 4.9)	1.9 (0.6; 3.9)	0.572	2.5 (0.8; 4.9)	1.9 (0.6; 3.9)	0.572

*Evidence of publication bias according to Egger’s test (p < 0.05).

^¶^Evidence from 14 studies.

Sex differences were not observed using Renaud’s (1970) technique. According to this technique, the most prevalent pattern was type C (> 12%), while type I was the least prevalent (< 1%) (Table 6).

**Table 6:** Lip pattern prevalence according to sex and dental arch for Renaud’s method for cheiloscopy classification.

	Left side using Renaud's (n = 3)^¶^			Right side using Renaud's (n = 3)^¶^		
	Male	Female	P value	Male	Female	P value
**Upper dental arch**						
Type A	12.7 (3.2; 26.9)	8.1 (0.2; 25.0)	0.622	8.0 (0.0; 29.5)	6.7 (0.0; 23.7)	0.889
Type B	8.5 (0.0; 29.7)	6.8 (0.0; 25.0)	0.867	12.2 (0.0; 42.0)	8.3 (0.0; 30.0)	0.783
Type C	12.6 (3.2; 26.8)	18.8 (10.6; 28.7)	0.439	12.4 (0.8; 34.2)	19.5 (8.2; 34.2)	0.542
Type D	5.2 (2.4; 8.9)	5.4 (1.1; 12.6)	0.952	6.9 (0.5; 19.4)	7.5 (0.9; 19.7)	0.922
Type E	8.0 (1.6; 18.6)	9.4 (4.3; 16.4)	0.796	9.6 (2.8; 19.8)	4.8 (0.2; 14.2)	0.399
Type F	2.2 (0.0; 10.4)	2.5 (0.0; 8.7)	0.937	1.3 (0.0; 5.3)	1.8 (0.0; 6.6)	0.840
Type G	15.3 (4.6; 30.6)	8.9 (2.9; 17.8)	0.399	7.3 (0.6; 20.3)	8.3 (1.9; 18.5)	0.892
Type H	11.3 (2.4; 25.2)	11.5 (0.7; 32.2)	0.979	11.9 (1.5; 30.1)	12.0 (0.9; 32.7)	0.999
Type I	0.0 (0.0; 0.3)	0.8 (0.0; 3.1)	0.166	0.0 (0.0; 0.3)	0.8 (0.1; 2.0)	0.048
Type J	9.0 (0.0; 34.2)	14.2 (0.1; 44.2)	0.736	13.0 (4.3; 25.3)	10.9 (0.0; 38.9)	0.867
**Lower dental arch**						
Type A	4.8 (0.0; 21.4)	4.8 (0.1; 24.8)	0.998	9.7 (1.5; 23.8)	5.8 (0.0; 25.2)	0.678
Type B	5.6 (0.0; 24.5)	9.0 (0.0; 29.7)	0.739	3.0 (00; 19.1)	6.7 (0.0; 26.9)	0.664
Type C	17.8 (5.0; 36.4)	22.5 (5.2; 47.1)	0.744	19.3 (5.3; 39.2)	27.0 (7.8; 52.8)	0.603
Type D	8.0 (5.0; 11.7)	6.2 (3.9; 8.9)	0.375	8.8 (3.8; 15.6)	5.2 (3.8; 6.7)	0.201
Type E	15.0 (3.2; 33.1)	18.3 (6.3; 34.8)	0.762	16.7 (3.5; 36.8)	17.9 (5.6; 35.0)	0.921
Type F	4.9 (0.0; 24.6)	3.7 (0.0; 13.5)	0.868	2.1 (0.0; 7.9)	2.6 (0.0; 9.3)	0.884
Type G	12.2 (4.6; 22.8)	8.9 (3.1; 17.3)	0.573	12.2 (5.2; 21.5)	8.8 (2.6; 17.9)	0.556
Type H	8.2 (0.3; 24.4)	7.5 (0.4; 21.8)	0.930	6.6 (1.2; 15.7)	7.9 (0.1; 24.9)	0.867
Type I	0.1 (0.0; 0.8)	0.1 (0.0; 0.7)	0.967	0.0 (0.0; 0.2)	0.3 (0.0; 1.7)	0.377
Type J	4.5 (0.0; 15.4)	4.6 (0.6; 11.8)	0.991	8.6 (5.2; 12.8)	4.9 (0.7; 12.2)	0.329

^¶^Evidence from 14 studies.

### Certainty of evidence

GRADE approach showed low certainty of evidence. The limiting aspects were the lack of consistency between the estimated effects and the lack of overlap of confidence intervals—evidenced by the increased heterogeneity between the included studies (Table 7).

**Table 7:** Grading of Recommendations Assessment, Development, and Evaluation (GRADE) summary of findings table for the outcomes of the systematic review and meta-analysis.

Quality assessment							Summary of results			Importance
N	Study Design	Methodological Limitations	Inconsistency	Indirectness	Imprecision	Other considerations	Number of participants	Effect	General quality	
								Accuracy (95% CI)		
“**Is cheilososcopy a reliable method for estimating sex?**”										
7	Diagnostic accuracy studies	Not serious^a^	Serious^b^	Not serious^c^	Serious^d^	none	1547	76.76 (65.81–87.70)	⨁⨁ LOW	Critical

GRADE Working Group grades of evidence.

High certainty: We are very confident that the true effect lies close to that of the estimate of the effect.

Moderate certainty: We are moderately confident in the effect estimate: The true effect is likely to be close to the estimate of the effect, but there is a possibility that it is substantially different.

Low certainty: Our confidence in the effect estimate is limited: The true effect may be substantially different from the estimate of the effect.

Very low certainty: We have very little confidence in the effect estimate: The true effect is likely to be substantially different from the estimate of effect.

^a^Majority of the studies presented low risk of bias; ^b^ The heterogeneity (I^2^) was high (> 75%) and no overlapping of effect estimates; ^c^ Evidence stems from an adequate population; ^d^ Wide credible confidence interval.

## Discussion

Dental analysis, within forensic dentistry, figures as an alternative for human identification especially because of the resistance of human teeth to high temperature and cadaveric alterations^85^. Over time, several forensic applications were studied for the use of dental/oral evidence. Apart human identification, bite mark analysis^86^ anthropological estimation of age^87^, sex^88^, stature^89^ and ancestry^90^; rugoscopy^91^ and cheiloscopy^92^ currently represent fields of forensic odontology. While some fields developed with strong scientific basis and broad legal acceptance (i.e. human identification), other fields remained controversial and lacked high-level evidence-based confirmation—this is the case of cheiloscopy. From the perspective of forensic practice, the alleged contribution of cheiloscopy relies on the possibility of retrieving identifying information (such as sex) from a suspect from visible or latent lip prints left in a crime scene^93^. Two main controversies might arise from cheiloscopy: (I) in crime scene investigations, the existing lip print left on objects or other surfaces could enable higher evidence toward human identification through DNA extraction instead of comparative analysis of furrows; (II) studies on cheiloscopy are generally observational, cross-sectional and with questionable settings that include different techniques, underlying surfaces and registration materials (e.g. lipsticks and powdered metals). In this scenario, several questions are pertinent: Why the scientific literature is so vast of studies on cheiloscopy for sexual dimorphism? How often is cheiloscopy used by forensic dentists in practice? But especially (claimed in many studies): Is cheiloscopy really useful to distinguish male and females in forensic dentistry?

To the present, there is no *antemortem* database of lip patterns worldwide (even in clinical dentistry). Moreover, registering the lips with photographs or other tools is rare—so, the application of cheiloscopy for human identification is limited from the beginning. Striving for sexual dimorphism could be an interesting asset to the armamentarium of forensic dentists, but again the application in practice is relative, especially because dental human identification is mainly necessary in challenging cases that involve charred bodies and skeletal remains^94^—in which lips are usually destroyed. Additionally, sexual dimorphism should be accomplished from body structures scientifically known for their anthropological reliability, namely the pelvic bones and skull^95^.

The evidence brought through the present systematic review was extracted from 72 studies that sampled 22,965 individuals. Out of the studies, 70% (n = 52)^1,4–6,10–15,25,27,30,31,35–40,43–46,48–50,53–57,59–62,64,66–75,78,79,81,83,84^ were from India. At first sight, the quality of studies was not bad when it comes to assessment of the risk of bias (nearly 70% had low risk of bias). These outcomes combined with the general quantification of the studies that detected sex differences based on lip pattern (67%) could lead to dangerous interpretations from readers that are not familiar with systematic reviews. A deeper look on the quantified outcomes of the most prevalent techniques (Suzuki & Tsuchihashi, 1970, n = 64, 88%; Renaud et al., 1973, n = 4, 5%), however, depicts an emerging lack of statistical significance (p > 0.05) for each lip pattern between males and females. The analysis performed per pattern clarifies the scenario as most of the studies in the field only test sexual dimorphism by comparing generalized (combined) patterns within sex groups (males vs. females). Further on, the limitations of cheiloscopy for sexual dimorphism is corroborated by GRADE assessment outcomes, which pooled seven studies (10% of selected studies) and 1,547 participants to clearly point out high heterogeneity (> 75%). The heterogeneity might be justified mainly because none of the 72 observational eligible studies reported data using scientifically established guidelines, namely STROBE. The resulting analysis via GRADE suggested low level of general quality and critical level of importance. Considering the diagnostic accuracy of cheiloscopy, mean outcomes point to 76%, which indicates that one in every four analysis of sexual dimorphism through lip patterns will have a wrong classification. Stronger outcomes would necessarily require a higher level of accuracy and a lower level of heterogeneity across studies. Summed up, the eligible studies screened and assessed in the present systematic review showed a good performance of cheiloscopy when the studies were analyzed separately; but when it comes to deeper analyses, especially observed per lip pattern within the techniques, lack of evident differences were detected between males and females. The limitation of cheiloscopy is, therefore, corroborated with the final quantitative assessment via GRADE.

To the present, the alleged contribution of cheiloscopy in forensic dentistry is merely superficial and highly relative. The quantification of the potential error within the diagnostic accuracy of cheiloscopy would be close to 25%—in other words, nearly 386 participants sampled in the quantitative part of this review would have their sex wrongly classified from a sample of 1547 individuals. Forensic dentistry itself is already a relative tool for human identification (not necessarily applicable in every single autopsy). In general, charred victims and skeletal remains consist of the main scenarios for a forensic odontologist. Authors might claim lip print applications to narrow disaster victim identification lists by sex, but in most of these cases bodies are not intact. If the case is somehow improving cheiloscopy studies in the future, authors are encouraged to design more advanced analyses of the morphology of the human lips to the point of having enough evidence to support the development of clinical databases and protocols for lip recording. From the perspective of forensic practice, this systematic review does not encourage the use of cheiloscopy as the sole tool for sexual dimorphism.

## Conclusion

After revisiting 72 eligible studies with a pooled sample of 22,965 individuals, this systematic review revealed weak foundations for the use of lip print analysis for sexual dimorphism in forensic dentistry. The pooled sampled reduced within the meta-analysis showed an average rate of wrong sex classification of nearly 25%. The studies were highly heterogeneous as none of them followed proper EQUATOR guidelines for structuring methods and reporting data. GRADE analysis confirmed the low certainty of evidence suggesting that cheiloscopy is not a reliable tool in practice when it comes to sexual dimorphism.

## Supplementary Information

Supplementary Information.

## Data Availability

The datasets generated during and/or analysed during the current study are available from the corresponding author on reasonable request.
