# Supplementary material for: The weak evidence of lip print analysis for sexual dimorphism in forensic dentistry: a systematic literature review and meta-analysis
Source: Sci Rep. 2021 Dec 17;11:24192. doi: 10.1038/s41598-021-03680-3 (PMC8683473; doi:10.1038/s41598-021-03680-3)
Supplement: Supplementary file 1 — Supplementary Information. [file 41598_2021_3680_MOESM1_ESM.docx]

**Appendix 1** – Studies excluded in the reading of the full texts and the reasons for exclusion, and full text studies not found (n=26).

|  | **Study excluded** | **Reason for exclusion** |
| --- | --- | --- |
| 1. | Kisin and Chanturiia, 1983 | Study not found |
| 2. | Vahanwala and Parekh, 2000 | Study not found |
| 3. | Molano et al., 2002 | Study not found |
| 4. | Mohamed et al.,2009 | Study not found |
| 5. | Misra et al., 2011 | Study not found |
| 6. | Abbasi et al.,2012 | Out of objective |
| 7. | Kulkarni et al., 2012 | Out of objective |
| 8. | Oliveira, 2012 | Review |
| 9. | Savignac, 2013 | Study not found |
| 10. | Murkey, 2013 | Study not found |
| 11. | Kaul and Kaul, 2015 | Study not found |
| 12. | Ballur et al., 2016 | Study not found |
| 13. | Chimurkar et al., 2016 | Study not found |
| 14. | Karaca et al., 2017 | Out of objective |
| 15. | Kumar et al., 2017 | Study not found |
| 16. | Babu et al., 2017 | Study not found |
| 17. | Kumaran et al., 2017 | Study not found |
| 18. | Maheshwari et al., 2018 | Study not found |
| 19. | Abbasi, et al., 2018 | Out of objective |
| 20. | Ahuja and Dahiya, 2018 | Study not found |
| 21. | Shah et al.,2018 | Study not found |
| 22. | Sahu et al., 2018 | Study not found |
| 23. | Priya, et al., 2019 | Out of objective |
| 24. | Kelasi and Gheena, 2019 | Study not found |
| 25. | Ravikumar and Darshan, 2019 | Study not found |
| 26. | Sagar et al., 2019 | Study not found |

**REFERENCES**

1. Kisin, M.V., Chanturiia, A.V. The lip prints as an object of complex expert investigation in case of personal identification. *Sud Med Ekspert*. **26**,21-23 (1983).
2. Vahanwala, S.P., Parekh, B.K. "Study of lip prints as an aid to forensic methodology." *Toxicol Forensic Med*. **17**,12-18 (2000).
3. Molano, O., Andrés, M., Gil, C., Humberto, J., Jaramillo, R., Andrea, J. Ruiz, G., Maritza, S. Estudio queiloscópico en estudiantes de la Facultad de Odontología de la Universidad de Antioquia. *Rev Fac Odontol Univ Antioq*. **14**,29-37 (2002).
4. Mohamed, S., Panchmal, P.S., Vijaya, H. "Chieloscopy-Can it be a tool for sex determination?." *Medico-Legal Update*. **9**,30-32 (2009).
5. Misra, A., Misra, D., Rai, S., Dadu, M., Khatri, M., Mallick, P. "Cheiloscopy: a useful adjunct to forensic identification-a study of 200 individuals." *Toxicol Forensic Med*. **28**,38-41 (2011).
6. Abbasi, M.H., Muhammad, A.M., Rana, M.A.R. "Cheiloscopic Variation among the Students of Avicenna Medical College Lahore." *Pak J Med Sci*. **6**,769-772 (2012).
7. Kulkarni, N., Vasudevan, S.D., Shah, R., Rao, P., Balappanavar, A.Y. "Cheiloscopy: A new role as a marker of sagittal jaw relation." *J Forensic Dent Sci*. **4**,6-12 (2012).
8. Oliveira, A.R.L.M. Identificação Humana pelas Características Labiais (Thesis). Universidade Fernando Pessoa (2012). Retrieved from htps://www.rcaap.pt/detail.jsp?id=oai:bdigital.ufp.pt:10284/3408
9. Savignac, C. Contribution of cheiloscopy in forensic odontology (Thesis). Université Paul Sabatier, Faculté de chirurgie dentaire, Toulouse. **1**,66-70 (2013).
10. Murkey, P. Assessment on different age group subjects to discover the most common lip print pattern type among males and females in central India. *J Datta Meghe Inst Med Sci Univ*. **8**,196-20 (2013).
11. Kaul, N., Kaul, V. "A study on lip print pattern, ABO blood group and their correlation in the population of western up belt." *J Evol Med Dent Sci*. **4**,10056 (2015).
12. Ballur, M.S., Priyanka, M., Dayananda, R. "A Study of Lip Prints among Kerala Population." Indian J Med Forensic Med Toxicol. 10(1):122-125 (2016).
13. Chimurkar, V.K., Ninave, S., Sharma, P., Ninave, S. "Cheiloscopy: A tool for personal and forensic identification." *J Indian Forensic Sci*. **38**,15-17 (2016).
14. Karaca, Z.B., Ayla, K., Ayca, U., Yener, C., Ozkan, O. Upper and Lower Lip Soft Tissue Thicknesses Differ in Relation to Age and Sex. *Int. J. Morphol*. **35**,852-858 (2017).
15. Kumar, L., Kumar, N., Shah, A., Bhuwnesh, S. "Forensic Anthropology-Lip Print Pattern in North Indians." J Punjab Acad Forense Med Toxicol. **17**,72-76 (2017).
16. Babu, U.R., Ameena, A., Imaad, M.I. "Can Lip Print Patterns Determine Sex of an Individual?." *Medico-Legal Update*. **17**,48-53 (2017).
17. Kumaran, S.M., Kumar, B.B., Lavlesh, K., Sweta, P.H. "Correlation between fingerprint and lip print pattern in Gujarati population." *Medico-Legal Update*. **17**,217-221 (2017).
18. Maheshwari, P., Gupta, P., Prakash, P., Jain, P., Raparia, P., Saxena, Y. "Correlation of lip prints with gender and blood groups." *J Indian Forensic Sci*. **40**,172-178 (2018).
19. Abbasi, M.H., Parveen, S., Gul, M., Khalid, U., Rehman, F., Kazmi, S.H., Khan, S., Arif, M.Z., Kalsoom, M., Sharif, Z., Malik, S., Hanif, T., Tariq, F. Lip Facsimilia Difference among the Students of RAI Medical College Sargodha. *Pak J Med Sci*. **12**,814-816 (2018).
20. Ahuja, P.T.P., Dahiya, M.S. "Cheiloscopy: A study on lip print patterns among the gujarati population." *Indian J Med Forensic Med Toxicol*. **12**,148-153 (2018).
21. Shah, U.P., Noor, F., Shah, Z.P., Ashai, F. "Study of lip print pattern amongst the students of nims medical college." *Indian J Med Forensic Med Toxicol*. **35**,39-42 (2018).
22. Sahu, G.J., Chandra, C., Debabratta, G. "Cheiloscopy: A Forensic Aid for Personal Identification and Sex Determination." *J Indian Forensic Sci*. **40**,10-16 (2018).
23. Priya, S.P., Anoud Salem, A.A., Thomas, S., Bhaskar, S., Alkhaldi, C.K.H., Al Samahi, H.A., Ahmed, A. Study on Lip Prints—To Estimate the Reliability as a Personal Identification Method. *World J Dent*. **10**,186-191 (2019).
24. Kelasi, P.S.A., Gheena, S. "The Study of Lip Prints in Relation to Gender and Facial form among Third Year Students in a Dental Institution”. *Indian J Med Forensic Med Toxicol*. **13**, 47-52 (2019).
25. Ravikumar, R., Darshan, C. "Cheiloscopy: The study of lip prints in sex determination." *Int J Med Toxicol Legal Med*. **22**,88-91 (2019).
26. Sagar, S., Sanat Kumar, B., Satya, R.M. "Evaluation of the Utility of Cheiloscopy, Rugoscopy, Dactyloscopy and Odonto-Morphometry for Human Identification and Gender Determination in an Eastern Indian Population: An Observational Study." *Indian J Med Forensic Med Toxicol*. **13**,1902-1907 (2019).
